# Restoration of Normal NF1 Function with Antisense Morpholino Treatment of Recurrent Pathogenic Patient-Specific Variant c.1466A>G; p.Y489C

**DOI:** 10.3390/jpm11121320

**Published:** 2021-12-07

**Authors:** Elias K. Awad, Marc Moore, Hui Liu, Lukasz Ciszewski, Laura Lambert, Bruce R. Korf, Linda Popplewell, Robert A. Kesterson, Deeann Wallis

**Affiliations:** 1 Department of Genetics, University of Alabama at Birmingham, Birmingham, AL 35294, USA; eliawad14@gmail.com (E.K.A.); huiliu@uabmc.edu (H.L.); ljack12@uab.edu (L.L.); bkorf@uab.edu (B.R.K.); kesterso@uab.edu (R.A.K.); 2 Centre of Biomedical Sciences, Department of Biological Sciences, Royal Holloway, University of London, Egham, Surrey TW20 0EX, UK; marc.moore@rhul.ac.uk (M.M.); Lukasz.Ciszewski.2008@live.rhul.ac.uk (L.C.); linda.popplewell@rhul.ac.uk (L.P.)

**Keywords:** neurofibromatosis type 1, cryptic splice, antisense oligo therapeutics

## Abstract

Neurofibromatosis type 1 (NF1) is an autosomal dominant genetic disorder with almost 3000 different disease-causing variants within the *NF1* gene identified. Up to 44% of these variants cause splicing errors to occur within pre-mRNA. A recurrent variant in exon 13, c.1466A>G; p.Y489C (Y489C) results in the creation of an intragenic cryptic splice site, aberrant splicing, a 62 base pair deletion from the mRNA, and subsequent frameshift. We investigated the ability of phosphorodiamidate morpholino oligomers (PMOs) to mask this variant on the RNA level, thus restoring normal splicing. To model this variant, we have developed a human iPS cell line homozygous for the variant using CRISPR/Cas9. PMOs were designed to be 25 base pairs long, and to cover the mutation site so it could not be read by splicing machinery. Results from our in vitro testing showed restoration of normal splicing in the RNA and restoration of full length neurofibromin protein. In addition, we observe the restoration of neurofibromin functionality through GTP-Ras and pERK/ERK testing. The results from this study demonstrate the ability of a PMO to correct splicing errors in *NF1* variants at the RNA level, which could open the door for splicing corrections for other variants in this and a variety of diseases.

## 1. Introduction

Neurofibromatosis type 1 (NF1) is an autosomal dominant genetic disorder characterized by the presence of cafe-au-lait spots, cutaneous neurofibromas, bone defects, optic nerve gliomas, and Lisch nodules. The *NF1* gene, located on the long arm of Chromosome 17, spans more than 282,000 bases of genomic DNA [1]. The gene constitutes 58 exons encoding a 12 kb mRNA transcript that creates a >250 kDa protein. This protein is expressed in a wide variety of tissues and functions as a tumor suppressor, by deactivating Ras through enhancement of its intrinsic GTPase activity [1].

Point mutations in the *NF1* gene can have varying effects on cellular mechanics and clinical presentations. Almost 3000 different disease-causing variants within the *NF1* gene have been identified in the Human Gene Mutation Database (http://www.hgmd.org, accessed on 22 November 2021), with up to 44% of these variants causing splicing errors to occur within pre-mRNA [2,3]. One study of 282 different *NF1* point mutations indicates that 44% of these mutations affect splicing, with 80% of those located in the consensus sequences [3]. The remaining 20% of splice variants are in deep intronic sites or in the coding region. While some involve the canonical GT splice donor or AG splice acceptor, many occur at less conserved positions within the 5′ or 3′ splice sites and still others create a novel 5′ or 3′ splice site [4]. These variants in the pre-mRNA can result in different transcriptional deficits, such as exon skipping, intron inclusion, or deletions of part of the exon.

**Figure 1:**
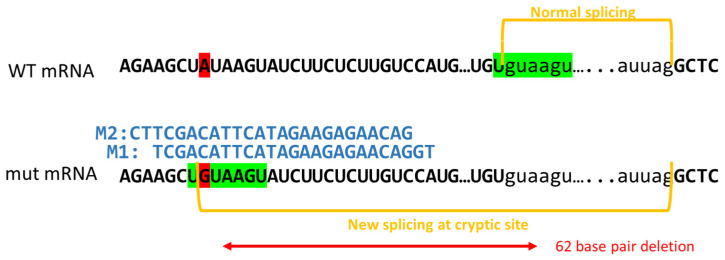
Pathogenic patient-specific variant c.1466A>G; p.Y489C alters splicing. Schematic of wild type (WT) and mutant exon 13 sequence surrounding c.1466. The top line depicts WT mRNA transcript with c.1466A highlighted in red. Exonic sequences are denoted by capital letters and intronic sequences are in lower case font. Normal spicing occurs at the canonical 5′ and 3′ splice sites denoted by “gu” and “ag” and is indicated with yellow brackets. The 5′ recognition sequence is highlighted in green. The mutant mRNA transcript is represented below with the A>G variant highlighted in red. Notably, this creates an alternative 5′ splice site denoted by the canonical “GU” sequence. The recognition sequence is again highlighted in green and is identical to that in the WT transcript. Yellow brackets again denote where splicing occurs and the resultant 62 base pair deletion that leads to a frameshift is indicated by a red arrow. Morpholinos M1 and M2 are also depicted above the variant transcript to show where they bind to mask the cryptic 5′ splice site.

A relatively common recurrent variant that has been reported 46 times in the Leiden Open Variation Database (LOVD) within exon 13, c.1466A>G; p.Y489C, forms a cryptic splice site, leading to aberrant splicing. This aberrant splicing results in a 62 base pair deletion from the mRNA and subsequent frameshift (Figure 1). While this is a relatively common recurrent *NF1* variant; the most recurrent *NF1* variant, R1809C, is reported in 75 unrelated patients [5]. Historically, RT-PCR was used to identify that c.1466A>G creates a new splice donor site which generates the formation of a stop codon at amino acid 489 (Figure 1) [4,6]. This frameshift leads to the inability of NF1 to deactivate Ras, resulting in an increase in GTP-Ras levels. Previously, we created a cDNA of this variant (Y489C) that was not subject to splicing and showed that the missense variant alone did not disrupt Ras signaling [7]. More recently, we have shown that the variant had no effect on neurofibromin’s expression, indicating that if the aberrant splicing could be corrected, the missense variant may not be deleterious [7,8]. Hence, a PMO approach might be feasible as a treatment strategy.

Antisense oligonucleotide (ASO) therapeutics can modulate splicing and be used to target variants like these. We have utilized phosphorodiamidate morpholino oligomers (PMOs) (a type of ASO), which are uncharged analogs of nucleic acids, usually ranging from 18–30 base pairs long. These strings of nucleic acids bind to complementary sequences of RNA, preventing cellular functions from occurring, such as blocking a splice site or repressing a splicing repressor. PMOs are built on a backbone of mopholine rings connected by phosphorodiamidate linkages and are resistant to a variety of enzymes. Antisense oligos are currently being used to treat diseases like B-thalassemia [9], Duchenne muscular dystrophy [10], and several other genetic conditions. Such oligos have also been evaluated for deep intronic *NF1* variants and were shown to restore normal splicing in primary fibroblast and lymphoblast cell lines bearing three different deep intronic variants (c.288+2025T>G; c.5749+332A>G; and c.7908−321C>G) [11]. ASOs targeted the newly created 5′ ss to prevent the incorporation of cryptic exons and were able to restore normal splicing, with rapid effects that lasted for several days. GTP-Ras levels were also decreased by the ASOs.

To model the Y489C variant, we have developed a human iPS cell line homozygous for the variant. We designed specific PMOs to block the cryptic splice site variant in exon 13, c.1466A>G. The results show that PMO treatment could restore normal splicing, neurofibromin protein levels, and neurofibromin function.

## 2. Materials and Methods

### 2.1. Mutant Cell Line Generation and Characterization

We utilized Synthego to generate knock-in pools of mutant iPSCs in the PGP1 parental background line. The CRISPR guide for Y489C was: ACTTATAGCTTCTTGTCTCCAGG. The Donor Sequence: CATGAATTAGTTTCACCATGGACAAGAGAAGATACTTATAGCTTCTTGTCTCCAGGTCTGTAGGTTTTTCTTTAAATTTAAGGCTTGTTACTTTTTCTTT. PCR primers were: Forward 5′ TGTATGTCTGGGGAATGTTTGT 3′ and Reverse 5′ GGCGTTTCAGCTAAACCCAATT 3’. Wild type (WT) and variant PGP1 cells were sent to our lab for further single cell selection, screening and detection. We single cell cloned the pools of targeted cells and isolated a single cell line that was homozygous null for Y489C out of 60 total clones. The variant was verified by sub-cloning and sequencing.

Human iPSC culture: Human iPSCs were maintained in mTeSR1 media on matrigel-coated tissue culture plates. Cells were passaged routinely with EDTA. Briefly, cells were washed twice with PBS-EDTA medium (0.5 mM EDTA in PBS), then incubated with PBS-EDTA for 5 min at 37 °C. PBS-EDTA was removed, and cells were washed off swiftly with a small volume of corresponding medium.

### 2.2. Cell Line Characterization

#### 2.2.1. Western Blotting

Cells were lysed with RIPA buffer and lysates were cleared by centrifugation at 20,000 RPM for 20 min at 4 °C. Protein was quantified with a Bradford assay and 50 μg of protein was loaded per well for NF1 blots, and 10 μg of protein was loaded for other blots. Eight percent SDS-polyacrylamide gels were run at 100 V for 2 h and transferred at 100 V for 2 h onto PVDF. Blots were probed overnight at 4 °C with primary antibody washed and probed 1 h at room temperature with secondary. Primary antibodies include N-Terminal NF1 (Cell Signaling cat# D7R7D 1:1000), actin (Cell Signaling cat# 4967 1:1000), p-ERK (Cell Signaling cat# 9101 1:1000), total ERK (Cell Signaling cat# 9102 1:1000), p-AKT (Cell Signaling cat# 4060L 1:1000), total AKT (Cell Signaling cat# 4691L 1:1000), pS6 (Cell Signaling cat# 2215S 1:1000), and total S6 (Cell Signaling cat# 2217L 1:1000). Secondary antibody was HRP-tagged from Santa Cruz. Chemiluminescent substrate from Bio-Rad was used as per manufacturer’s protocols.

#### 2.2.2. RAS-G-LISA Assay

The RAS-G-LISA assay was obtained from Cytoskeleton Inc. and was performed according to the manufacturer’s instructions. iPSC cells were grown in mTESR plus media and plated into 6 well plates coated in matrigel for adherence, and left to grow overnight. Cells were treated with or without morpholino and control oligo for 48 h. They were then washed with ice cold PBS and lysed with Cell Lysis Buffer for protein extraction. Lysates were cleared by centrifugation and immediately snap frozen. Protein content was quantified by BSA and concentrations equalized among the samples with the addition of Lysis Buffer. Ras protein was used as the positive control and blank samples were used for negative controls. The Cytoskeleton GTP-Ras binding plate was used and cell lysates and controls were added and incubated. Active, GTP-bound Ras in cell lysates will bind to the wells, while inactive GDP-bound Ras is removed during washing steps. The bound active Ras is detected with a Ras-specific antibody, with an HRP-conjugated secondary antibody. Absorbance was measured at 490 nm using a microplate spectrophotometer.

### 2.3. PMO Design and Treatment

PMOs were designed in accordance with previously published work [12]. Briefly, *NF1* exon 13 cryptic splice site was analyzed. PMOs were designed to physically bind and mask the site. Morpholino sequences include: M1: TGGACAAGAGAAGATACTTACAGCT; M2: GACAAGAGAAGATACTTACAGCTTC; and Control (Ctrl): CCTCTTACCTCAGTTACAATTTATA.

Custom morpholinos were ordered from Gene Tools, LLC and reconstituted in 0.22 µM filtered ultrapure nuclease-free water, to make a 1 mM stock concentration, as per the manufacturer’s directions. WT or Y489C-containing PGP1 iPS cells at ~70–80% confluence were treated with PMOs diluted in 1 mL of fresh culture media at desired concentrations, alongside 6 µM of Endo-Porter (DMSO) (Gene Tools LLC). Cells were incubated at 37 ℃, 5% CO_2_ for 48 h prior to harvest.

### 2.4. PMO Efficiency Assay

Post-treatment with PMOs, cells were lysed in RLT buffer and total RNA extracted using an RNeasy Miniprep Kit (Qiagen, Manchester, UK). Extracted RNA was subject to a MMLV mutant cDNA synthesis reaction (ThermoFisher Scientific, Waltham, MA, USA). Briefly, 0.5 µg RNA was added to 1 µL 50 µM Oligo d(T)20, 1 µL 10 mM dNTP mix, and made up to 14 µL with nuclease-free water; this RNA-water mix was heated to 65 °C for 5 min. Then, a combination of 4 µL 5× SSIV RT Buffer, 1 µL 100 mM DTT, 1 µL RNaseOUT Recombinant RNase Inhibitor, and 1 µL Superscript Reverse Transcriptase were added to the RNA mix. RT reaction mix was then incubated at 55 °C for 10 min, and then inactivated at 80 °C for 10 min. Resultant cDNA was then amplified via PCR. Amplification was achieved with Phusion High-Fidelity DNA Polymerase (New England Biolabs, Ipswich, MA, USA). The standard 20 µL PCR reaction contained 50 ng of cDNA and was comprised of Nuclease-free Water, 0.5 µM forward and reverse primers, 200 µM dNTPs, 1× Phusion HF Buffer, and 0.02 U/uL Phusion DNA Polymerase. Primers used were: Forward 5′ GAATGGCACCGAGTCTTAC 3′ and Reverse 5′ CAGCAGAGCCTCCATTGCTT 3′, yielding a full length amplicon of 169 bps and an exon 13 deletion amplicon of 107 bps. Thermocycling conditions: 98 °C for 30 s, 35× cycles [98 °C 10 s, 54 °C 30 s, 72 °C 30 s] and 72 °C for 8 min.

A 20 µL aliquot of the PCR product was resolved on a 3% *w/v* agarose in TAE buffer at 70 V for 2 h and imaged. The images were then subject to densitometric analysis using ImageJ software. Arbitrary values assigned to amplicons based upon analysis were then subject to the following calculation.

% Normal Splicing = (Normal splice transcript)/(Total Product (Normal + Variant Splice Transcript)) ∗ 100

### 2.5. Statistical Analysis

All assays were repeated a minimum of three times. Error bars represent standard deviation (SD). Statistical comparisons were made using Student *t*-test in Excel software to determine which results were statistically significant with *p* < 0.05.

## 3. Results

**Figure 2:**
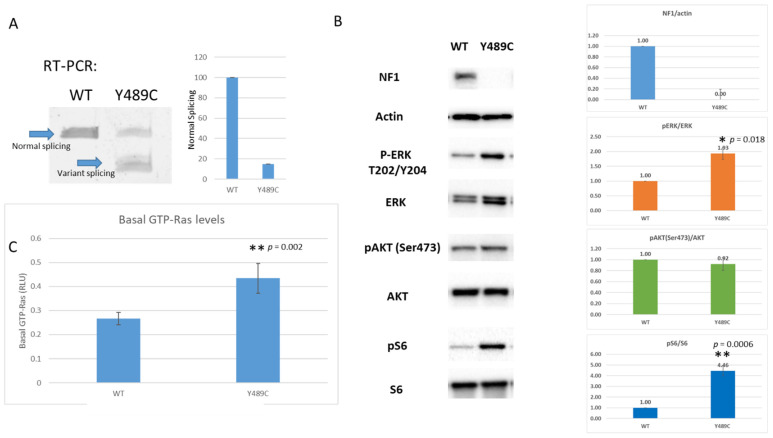
Characterization of c.1466A>G: p.Y489C variant specific iPS Cell Line model in comparison to WT parental PGP1 cells. (**A**) RT-PCR products on agarose gel, indicating leaky splicing of the variant. Quantitation of normal and abnormal splice variants indicates ~15% normal splicing, as indicated by the histogram (Error bars represent SD; N = 6 experiments). (**B**) Representative Western blots of WT PGP1 cells and Y489C variant containing PGP1 cells. Indicated Ras-pathway antibodies show altered Ras-signaling in the variant cells. Histograms to the right of the blots show quantification of N = 3 independent experiments. Error bars represent SD. Asterisks indicate statistical significance (* *p* < 0.05; ** *p* < 0.01). (**C**) Histogram evaluating GTP-Ras levels in WT and variant-containing cells.

CRISPR-Cas9 was utilized to edit iPSC lines to more accurately model this variant. iPSCs were chosen to model the variant as NF1 phenotypes affect numerous different cell types. *NF1* loss of heterozygosity (LOH) in the Schwann cell lineage leads to neurofibroma development, *NF1*^−/−^ melanocytes lead to café-au-lait macules and Lisch nodules, *NF1*^−/−^ osteoblasts lead to pseudoarthrosis of the tibia, and *NF1*^−/−^ in glial cells leads to astrocytomas. Hence, iPSC may allow us the study the effects of the same variant with the same background in multiple cell types upon future differentiation. Single cell clones were isolated from pools of cells targeting the exon of interest. Only one clone positive for Y489C was isolated and characterized (Figure 2). RT-PCR products indicate leaky splicing, with a normal sized splicing band representing about 15% of the total transcript and a variant band approximately 85% of the total transcript, compared to 100% WT transcript in WT cells (Figure 2A). Both RT-PCR product bands were sequenced to validate the expected products; the variant band recapitulates the 62 bp deletion due to cryptic splicing and the normal length band contains the Y489C missense variant (Supplemental Figure S1A). WT sequence is not present in the mutant cells. Despite the presence of normal length transcript containing the Y489C variant, total loss of neurofibromin protein expression is observed by Western blot (Figure 2B and Supplemental Figure S1B). Ras activity was also evaluated based on GTP-Ras levels, pERK/ERK ratios, pAKT/AKT ratios, and pS6/S6 ratios. This variant behaves as anticipated for the loss of NF1 function. This cell line has increased GTP-Ras levels over a WT cell line (*p* = 0.002) (Figure 2C). Markers of downstream Ras signaling are increased in comparison to WT cells, as we see increased pERK/ERK (*p* = 0.018) and pS6/S6 levels ratios (*p* = 0.0006). pAKT/AKT levels remain unchanged (*p* = 0.764).

**Figure 3:**
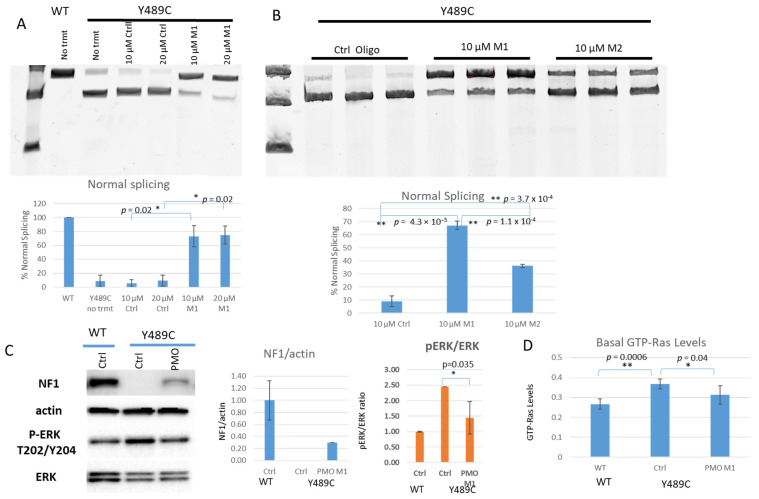
Treatment of Y489C cells with PMOs restores splicing, NF1 protein expression, and function. (**A**) RT-PCR products on representative agarose gel for WT and Y489C containing cells treated with indicated doses of control (Ctrl) and M1 PMO of interest. Histogram below shows quantitation of normal sized transcripts as a function of percentage of total transcript. Notably, M1 is able to significantly restore splicing at both 10 and 20 µM. Error bars represent SD; N = 3 independent experiments. (**B**) RT-PCR products on agarose gel for Y489C containing cells treated with 10 µM dose of Ctrl, M1, and M2 PMOs of interest. Histogram below shows quantitation of transcripts. Notably, 10 µM M1 and M2 are both able to significantly restore splicing, though M1 is significantly more efficient. Error bars represent SD; N = 3 independent experiments. (**C**) Representative Western blot indicating NF1, actin, pERK, and ERK levels of WT and Y489C cells after treatment with Ctrl or PMO M1 at 10 µM. Histograms depict quantification of N = 3 independent experiments; error bars represent SD. PMO M1 is able to restore NF1 protein expression and lower pERK/ERK ratios. (**D**) Basal GTP-Ras levels for WT and Y489C cells after treatment with Ctrl of PMO M1. Asterisks indicate statistical significance (* *p* < 0.05; ** *p* < 0.01).

PMO Design and Testing: We designed two PMOs (M1 and M2) to bind directly to the variant (Figure 1). RT-PCR results of untreated WT and mutant cells, PMO control (Ctrl) treated mutant cells, and PMO M1 treated mutant cells are depicted in Figure 3A. WT cells show the normal-sized transcript. Mutant cells show both the aberrant transcript and small amounts of the normal sized transcript when untreated or treated with Ctrl oligo. The treatment of mutant cells with the targeted PMO M1 restores WT transcripts to ~70%, leaving ~30% aberrant transcript (*p* = 0.002 for both 10 µM and 20 µM). Next, we compared both M1 and M2 directly at 10 µM in three independent biological replicates, with a representative gel shown in Figure 3B. While M1 can restore normal splicing to 67% of transcript (*p* = 4.3 × 10^−5^ in comparison to Ctrl), M2 is only able to restore normal splicing to 36% of transcript (*p* = 3.7 × 10^−4^ in comparison to Ctrl). The difference between M1 and M2 is significant (*p* = 1.1 × 10^−4^); hence, all additional experiments focused only on PMO M1. Restoration of NF1 protein expression was restored only in M1-treated mutant cells, and not Ctrl-PMO treated mutant cells (Figure 3C and Figure S2). PMO M1 was able to restore approximately 30% of the levels of NF1 protein that were detected in WT cells. We also see restoration of Ras signaling inhibition, as treatment with the PMO M1 (and not the Ctrl oligo) is able to repress both pERK/ERK levels (*p* = 0.035) and GTP-Ras levels (*p* = 0.041) (Figure 3C,D and Figure S2).

## 4. Discussion

Overall, we show that the utilization of morpholinos can mask the c.1466A>G cryptic splice site and restore splicing at the normal site. We tested two different oligos, and M1 performed better than M2. Despite relatively high doses of PMOs, we did not observe complete suppression of use of the cryptic splice site in the iPSC line created. Normal splicing was restored to ~70% of total transcripts in the cells. Despite the prevalence of normalized splicing and production of transcripts harboring the missense variant, we see the restoration of only approximately 30% of normal neurofibromin protein levels. There is a disconnect between the level of full-length transcript generated upon PMO treatment, and the amount of neurofibromin protein produced in the cell. Based on recently published data, *mNf1* cDNA containing the Y489C missense mutation was able to produce a stable protein product [8], and thus, we suspect that this disconnect is not the result of a protein stability issue, but rather some other mechanism.

We developed an induced pluripotent stem cell line recapitulating a patient-specific *NF1* variant, in efforts to evaluate if antisense therapeutics could be feasible for the correction of an intragenic cryptic splice site. After CRISPR/Cas9 knock-in, the cell line appears nullizygous for the variant. While we performed subcloning and sequencing around the region, it is possible that the second allele contains a larger deletion that is not detectible via PCR cloning and sequencing. These cells appear to have leaky splicing at the cryptic splice site, as both normal length and deletion transcripts are detected via RT-PCR and sequencing. Notably, the normal length transcript does contain the Y489C missense variant, and is not the wild type allele. While we see presence of normal length RNA transcript with Y489C variant, there is a complete absence of neurofibromin protein expression on Western blots.

Loss of neurofibromin is known to upregulate Ras signaling, and we were able to demonstrate that this occurred in the novel cell line established. The Y489C PGP1 iPSCs show upregulated GTP-Ras, pERK/ERK and pS6/S6, but do not show altered pAKT/AKT. While it may have been anticipated that pAKT would be elevated in the iPSCs, it is unclear if unaltered pAKT/AKT is cell type- or variant-specific. It has recently been shown that Ras effector pathways can be cell type-specific [13]. GTP–Ras binds to numerous effectors to trigger various signaling cascades, which in turn modulate different cellular processes ranging from cell growth, survival, cell migration, differentiation, and death. The growing family of Ras effector proteins includes: RAF, PI3K, RalGDS and p120GAP, Rin1, Tiam, Af6, Nore1, PLCε and PKCζ [14]. There are distinct roles for the Ras effector pathways in regulating brain neural stem cell function (NSC) [13]. These studies used a combination of *Nf1* genetically engineered mice and pharmacological/genetic inhibition approaches to show that neurofibromin differentially controls NSC proliferation and multi-lineage differentiation through the selective use of the PI3K/AKT and RAF/MEK pathways [13]. While PI3K/AKT governs neurofibromin-regulated NSC proliferation, multilineage differentiation is MEK-dependent. These results indicate that PI3K-AKT and RAF-MEK pathways are both regulated by NF1, but lead to different cellular functions. It is possible that the relative abundance or availability of these different effectors results in cell type-specific phenotypes associated with NF1. In contrast, the existence of genotype-phenotype correlations with mild or severe phenotypes also argues that there can be variant-specific effects [5,15,16,17,18,19]. Further, as S6 is downstream of AKT, we were surprised to see dramatically elevated pS6 levels for the Y489C mutant cells, with essentially no difference in pAKT. It is likely that S6 is activated through another pathway. This has already been shown to occur in other neuronal disease models, such as the fragile X mouse neocortex, which shows that elevated ERK activity causes the overactivation of p90-ribosomal S6 kinase (RSK) and the hyperphosphorylation of ribosomal protein S6 [20].

While for some diseases, the restoration of even minimal levels of protein function may be therapeutic, it is not known how much neurofibromin might be required to restore a normal or near-normal phenotype [21]. There are multiple mitigating factors and data that influence our thinking about the protein level required for clinical improvement. First, affected individuals with the same mutation, including individuals in the same family, may differ in phenotypic severity, suggesting that the same level of neurofibromin restoration may have different therapeutic effects in one patient versus another, even in the case of two individuals with similar genetic background and identical *NF1* mutation. Secondly, different heterozygous patient mutations lead to different levels of expression of mutant and normal *NF1* alleles and, consequently, different (combined mutant and wildtype) neurofibromin protein levels within patient fibroblasts, ranging from 12% to 89% of normal levels [22]. Unfortunately, protein expression levels do not equate to function, as significantly elevated Ras activity (based on 2–3 fold increased levels of RAS-GTP) was observed across the entire range of neurofibromin levels in these fibroblasts [22]. Thirdly, as described in Li et al. 2016 [23], *Nf1*+/G848R mouse embryonic fibroblasts (MEFs) appear to have normal levels of neurofibromin (100%), while homozygous null G848R MEFs have only about half of the normal levels, resulting in a ~2.5-fold increase in p-ERK compared to wild type cells. Interestingly, this is similar to the effect of another heterozygous knockout allele, *Nf1*+/Δ4, which also has 50% neurofibromin levels and 2.5-fold increased p-ERK. Despite the reduction in neurofibromin and increase in pERK, homozygous G848R mice are phenotypically normal. This suggests that the restoration of at least 50% neurofibromin function may rescue some *in vivo* phenotypes but not pERK phenotypes, indicating that outcomes of molecular (or other) endpoint assays may differ depending on the phenotype in question. Fourthly, NF1 phenotypes are cell type-specific, and so are *NF1* expression levels (data available on GTEx Portal). Consequently, the amount of functional neurofibromin required may differ based on cell type. A recent study of homozygous G848R mice under glial fibrillary acidic protein (GFAP)-cre [24] found that, in brainstem astrocytes, 60% of (mutant) neurofibromin is present, and is enough to maintain many *in vivo* phenotypes that other *Nf1* loss of function mutations cannot. Fifthly, neurofibromin has been shown to form dimers [25,26], but depending on the mutation, a mutated neurofibromin may not be able to form dimers, not even with wild-type neurofibromin. On the other hand, a mutated neurofibromin can affect the functionality of the dimer. In the worst case, mutated neurofibromin sequesters functional neurofibromin, obtained from a normal allele, into non-functional dimers. The level of functional dimers needed for a normal or near-normal phenotype is not known, but may again depend on the cell type. Lastly, neurofibromin may well have other functions currently unknown that are not captured by existing assays. Hence, it is also not yet possible to determine how much neurofibromin is required for these unknown functions. Future steps will involve the development of an *in vivo* model system in which to test our PMO and a delivery vehicle, to ensure it reaches the cell types of interest at the correct time and at efficient levels. Importantly, we demonstrate that some *NF1* patient mutations are amenable to PMO-based therapeutics as we continue to overcome many of the challenges and unknowns to implement mutation-directed therapeutics for NF1.

## Data Availability

Data available on request from the authors.

## Supplementary Materials

The following are available online at https://www.mdpi.com/article/10.3390/jpm11121320/s1, Figure S1: Additional Characterization of c.1466A>G: p.Y489C variant specific iPS Cell Line. (A) Chromatograms of WT RT-PCR product from WT cells (top), normal length product from Y489C cells (middle) and deletion product from Y489C cells (bottom). (B) Replicate Westerns for Figure 2B from 3 independent experiments showing characterization of Y489C homozygous mutant iPS cells in comparison to WT iPS cells. Antibodies are indicated at the left, Figure S2: Replicate Westerns for Figure 3C from 3 independent experiments showing characterization of WT cells treated with STD control PMO and Y489C homozygous mutant iPS cells treated with STD control PMO and PMO M1. Antibodies are indicated at the left.
