# Supplementary material for: Restoration of Normal NF1 Function with Antisense Morpholino Treatment of Recurrent Pathogenic Patient-Specific Variant c.1466A>G; p.Y489C
Source: J Pers Med. 2021 Dec 7;11(12):1320. doi: 10.3390/jpm11121320 (PMC8705852; doi:10.3390/jpm11121320)
Supplement: Supplementary file 1 [file jpm-11-01320-s001.zip › jpm-1432915-supplementary.pdf]

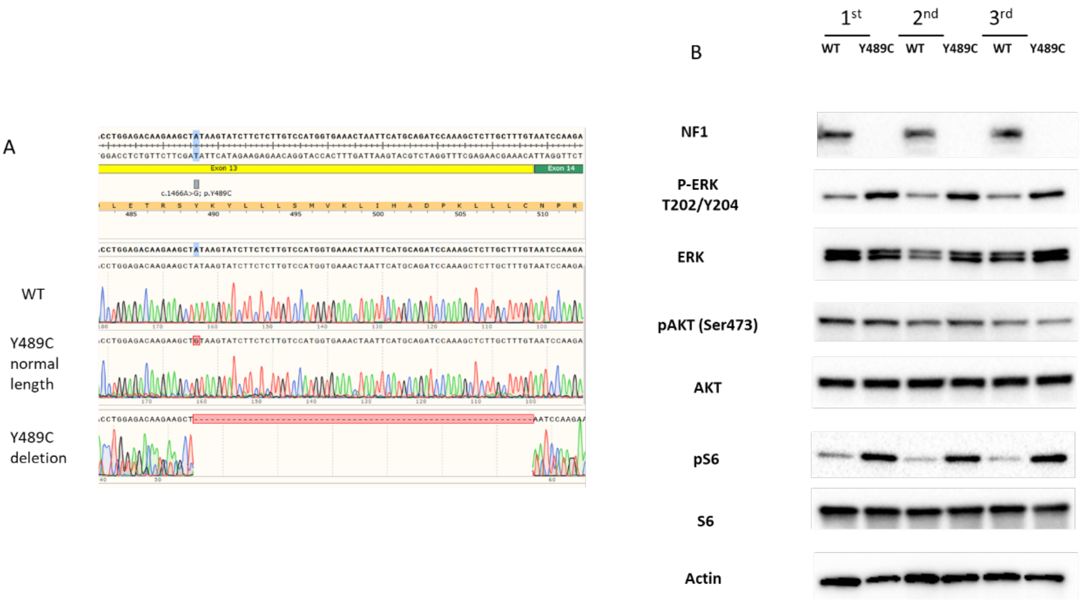

Supplemental Figure S1. Additional characterization of c.1466A>G: p.Y489C variant specific iPS cell line. A. Chromatograms of WT RT-PCR product from WT cells (top), normal length product from Y489C cells (middle) and deletion product from Y489C cells (bottom). B. Replicate Westerns for Figure 2B from 3 independent experiments showing characterization of Y489C homozygous mutant iPS cells in comparison to WT iPS cells. Antibodies are indicated at the left.

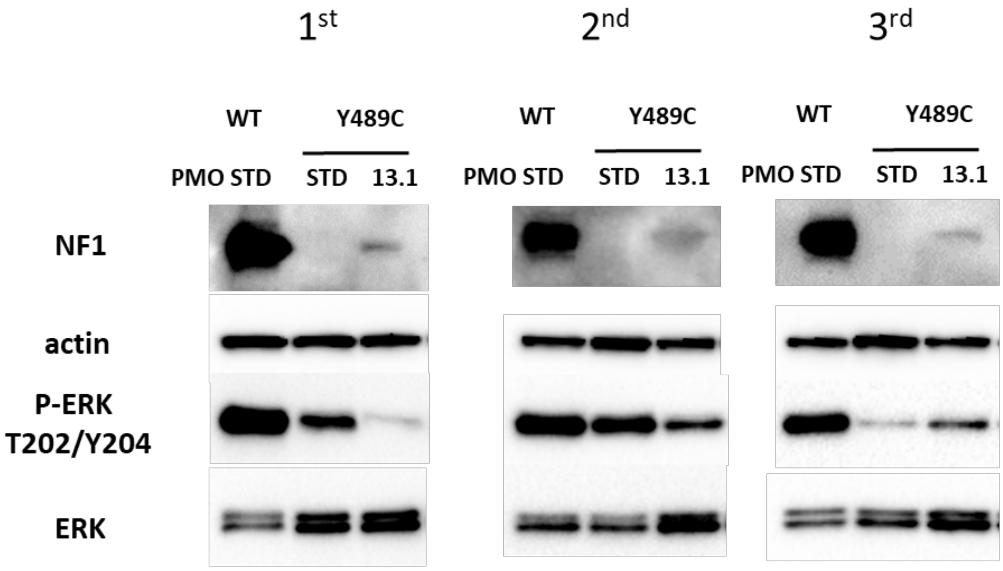

Supplemental Figure S2. Replicate Westerns for Figure 3C from 3 independent experiments showing characterization of WT cells treated with STD Ctrl PMO and Y489C homozygous mutant iPS cells treated with STD Ctrl PMO and PMO M1. Antibodies are indicated at the left.
